# The Instrumented Indentation Test: An Aiding Tool for Material Science and Industry

**DOI:** 10.3390/ma16145078

**Published:** 2023-07-19

**Authors:** Giovanni Maizza, Dongil Kwon

**Affiliations:** 1 Department of Applied Science and Technology, Politecnico di Torino, 10129 Torino, Italy; 2 Department of Materials Science and Engineering, Seoul National University, Seoul 08826, Republic of Korea; dongilk@snu.ac.kr

## 1. Foreword

Engineering materials encompass a wide spectrum of structural–functional products that are commonly used in the transportation (automotive, aerospace, naval), construction, machinery, and tooling industries. The final shapes and performances of such products are determined by a sequence of primary and secondary manufacturing processes. In many cases, the performances are intimately linked with and depend on a complex self-balanced (elastic) residual stress state which may enhance or weaken the expected performances of the products in relation to the target application [1].

The quality and performance of engineering products are normally assessed at the end of the manufacturing route via standard testing methods, which are generally of a destructive type (e.g., macro-hardness, tensile test, impact test, and so forth). However, these methods are not capable of discerning between residual stresses and the intrinsic properties of a material. X-ray diffraction techniques are used, although not so frequently, to measure the residual stresses over the surface of engineered products at the microscopic scale [1].

The employment of a quick, viable, non-destructive assessment method, which can be used not only at the end of the manufacturing process but also at all the critical steps of such a process, would introduce considerable benefits to industry in terms of improving the processes, quality, and reliability of the final products.

## 2. Summary of the Special Issue

The aim of this Special Issue is to show how the instrumented indentation test (IIT) can introduce unprecedented benefits to research and industry because of its quick, non-destructive, and low-cost features. Moreover, unlike the tensile test, only relatively small probing samples are required. The current state of the art is presented in a number of selected illustrative case studies. Further, the possible limits and shortcomings have also been reported. The following major benefits of ITT have been identified in the vast sector of engineering materials and their manufacturing: (a) the rapid setting up of the manufacturing process, (b) the identification of the critical manufacturing stages, such as the replacement of worn tools, (c) in-line process control through the monitoring of any relevant indentation properties at the desired depth of a product, (d) in-service structural health monitoring, (e) the determination of the tensile-like properties of a product (through a macro-instrumented indentation test, MIIT), and (f) the possibility of detecting subsurface biaxial residual stress induced by the manufacturing process. The current ISO 14577 standard, Part 1 to Part 4 [2,3,4], refers to bulk and coated materials, and it regulates the procedures used for testing, the construction of testing equipment, as well as the selection and preparation of test samples. It is worth noting that it strictly holds whenever the tested materials exhibit a pure elastic contact upon unloading. Furthermore, it does not cover the pile-up or sink-in effects naturally experienced by the majority of engineering materials. 

IIT is already successfully utilized in the industrial sector of coatings, thin films, and MEMS at the nanoscale (i.e., nanoindentation, nIIT) for final quality assessments [5], although it is used mostly in research and university laboratories for material research and nanoscale plasticity studies on metallic materials (see, for instance, [6,7]). Since its inception 40 years ago, nIIT has greatly progressed in technology, software, and measurement accuracy, thereby enabling the newest devices to run entire two-dimensional indentation mappings of hundreds of indentations over a short period of time, which in turn has led to its throughput in research and industry being maximized [7]. It has been claimed that the newly available operation mode of continuous stiffness measurement (CSM) allows a more accurate measurement of the indentation modulus to be achieved [8], although its application to engineering materials requires caution as dynamic testing may alter the existing self-equilibrated residual stress state across a sample.

A number of scientists, users, developers, engineers, and technical experts from the academic, industrial, and research areas have contributed to this first volume of the Special Issue on The Instrumented Indentation Test: An Aiding Tool for Materials Science and Industry. The present state of the art on the subject indicates that, in spite of promising experiences which encourage the transfer of IIT from the laboratory to industry [9,10,11,12,13,14,15,16,17,18,19], other factors currently act as an obstacle to its use (see, for instance [14,16]). 

The article by Schiavi et al. [9] is specifically addressed to new users who are experiencing the IIT method for the first time. The work is authored by expert metrologists who have used a primary hardness standard device that is routinely employed as a “reference tool” to determine the accuracy class of commercial hardness testers. The proposed study serves as a benchmark for the tested engineering materials. It is based on calibration procedures that are suggested in the ISO 14577 guidelines [2,3,4]. It focuses on the indentation modulus, work, hardness, and creep in the case of four (Fe-, Al-, Cu-based) engineering alloys that have been tested under macro-loading (MIIT) conditions using a Vickers indenter. A correlation is reported between indentation hardness and conventional Vickers hardness.

The comprehensive review by Montanari and Varone [10] offers net evidence on the simplicity, indentation size effect-free, roughness-insensitivity, and versatility features of flat-punch macro-indentation (FIMEC). These features are all very desirable for industrial applications. The article reports on the numerous mechanical properties (e.g., yield stress, Young modulus, ductile-to-brittle transition temperature, surface creep, and stress relaxation) that have been assessed over the past two decades and which have involved minute monolithic as well as composite material samples produced via a variety of processes (e.g., irradiation, welding, and deposition). The technique has also been proven effective under low- and high-temperature testing conditions.

The paper by Scales et al. [11] presents the behavior of spherical MIIT when applied as a non-destructive on-field testing method to segments of natural gas pipelines. The research was prompted by industry to comply with US federal regulations on the safeguarding and integrity monitoring of in-service pipelines. A portable indenter device was employed in this research. The authors attempted to investigate the correlation between the chemical composition and tensile-like properties of steels while assessing the possible deterioration events of the steels used in pipelines during service operation. Machine learning techniques were implemented to manage the massive amount of on-field recorded data.

Residual stresses are ubiquitous in most manufactured products and components. As compressive and/or tensile residual stresses typically induce pile-up and sink-in phenomena respectively during IIT, accurate prior test calibrations and ad hoc corrections are needed. At present, the influence of residual stresses on IIT properties is not fully understood (see for instance, [1]). 

The papers by Akatsu et al. [12] and Lee et al. [13] point out the crucial problem of residual stresses in engineering materials. The former attacked the problem computationally using a 3D FEM conical indentation model, specifically addressing the effect of pre-existing equi-biaxial pre-stress on indentation properties. The attained results show that the extent of pile-up and sink-in depends on the value of the Young’s modulus-to-yield stress ratio of the material. 

The study by Lee et al. is instead based on an experimental approach. They used the macro-instrumented indentation test (MIIT) to assess the performance of three arc-welded T-joints made of HY-grade (30 mm thick plate) vessel steels. The necessary free-stress region in the samples was associated with the electro-discharge machined regions that were liberated after the dissection operations of the joint samples. They measured the residual stress profiles along the fusion line on the top and the bottom of the plate surfaces using a method based on force difference measurements. The achieved MIIT-based residual stress profiles were successfully validated against the X-ray diffraction-measured counterparts at an appreciable level of confidence. 

The mechanical characterization of dissimilar welded joints is a complex problem of industrial and scientific relevance. The accurate inspection of the complex inter-phase mixture region, which develops when welding the two dissimilar materials in contact, determines the actual mechanical strength and toughness of a joint. 

The work by Maizza et al. [14] presents the results of the application of nITT to a dissimilar high-speed steel/WC-Co butt-welded joint produced by means of a solid-state capacitor discharge welding process. High-speed automated nIIT surveys enabled a detailed characterization of the full heat-affected region and also helped discern the mechanical properties of the individual phases in the multicomponent/multiphase regions. The results show how the achieved pointwise correlation between the microstructure and mechanical properties can help one find optimal welding parameters to ensure the optimal combination of strength and toughness of a welded joint.

The fundamental study by Pero et al. [15] on the nIIT properties of a WC-Co composite elucidates and confirms the special correlation that exists between the indentation properties and the orientation of the WC single crystal embedded in the Co matrix of a two-phase WC-10Co composite. Automated nIIT surveys allowed a new and more efficient evaluation method to be defined in order to search for the above correlation. This research approach is particularly important for the cutting tool industry, among others. 

The research by Cabibbo [16] was driven by the inconsistencies encountered in the application of the ISO code [2,3,4] to engineering materials. It points out the crucial importance of prior calibrations before performing nIIT to determine the properties of materials. He proposed an alternate calibration procedure that has been validated in the context of a large-scale EU round-robin research project. The aim of this project was that of minimizing the dispersion of IIT raw data from different commercial nanodevices, calibration methods, and post-processing software in order to attain the final indentation hardness and modulus of selected coating materials with improved accuracy.

The proposed calibration mainly focuses on the indenter geometry (area function) and machine compliance; three reference indenters and three reference materials were considered. The results shown in this work attest a reduced spread of the mean values of the determined indentation hardness, hence permitting a more accurate characterization to be made of individual microstructure features, such as twins in copper, carbides in 100Cr6 steel, pearlite globules, and ferrite grains in gray cast iron. 

nIIT plays an important role in expanding the science of elastoplastic materials in various forms (bulk, thin films, multilayer, coating, etc.), due to the possibility of using very small loads with a point indenter. 

The demand for the determination of a more accurate indentation modulus and contact area during nIIT led Huen et al. [17] to develop a novel inverse algorithm based on continuous stiffness measurement (CSM) data. This algorithm combines stiffness-based functions, derived from both dimensional analysis and computer simulation, and takes into account pile-up and sink-in profiles. It has been shown that the developed algorithm can derive the elastic modulus and hardness with improved accuracy, compared to the Oliver and Pharr method [2], as it is free of any scatter of the plasticity parameters, and does not need any prior best-fitting procedures, as requested in the ISO 14577 standard [2] over the investigated yield stress/indentation modulus ratio range.

This Special Issue also received contributions that are devoted to material science research.

The work by Ohmura and Wakeda [18] involves a comprehensive review on ex situ and in situ observations, including computer modeling on plasticity-induced phenomena in metals and alloys, especially during their incipient (pop-in) stage under point-indenter nano-indentation. The influence of superficial native oxide films and that of the initial defects, which can affect the mechanisms of dislocation nucleation beneath the indenter, are discussed in detail. Various unveiled phenomena, associated with dislocation nucleation and their activation mechanisms, have been illustrated in relation to several materials to examine the yielding phenomena in an attempt to shed light on the grain-boundary-induced strengthening mechanism. 

Finally, the possibility of efficiently automating nIIT has enabled developers to enhance the speed of indentation data acquisition and storage to a very high level. This has encouraged researchers to develop more efficient numerical methods to manage and post-process such data. For instance, Kossman and Bigerelle [19] took advantage of artificial intelligence and computer vision models to discriminate the presence of pop-in in a large number of recorded indentation curve data sets. The developed convolutional neural network model achieves an accuracy of 93% for the classification stage while efficiently sorting the P-h curves.

## 3. Conclusions

All of the articles submitted to this Special Issue deal with the latest key advance in the use of IIT in industry and research and pave the way toward a successful transfer of IIT to the vast industry of engineering materials and inherent manufacturing. The contributions have touched on various aspects of IIT, from a pedagogical basis to nanoplasticity science in metallic materials, while presenting important case studies of industrial applications with useful hints for new IIT developments and valuable suggestions for the revision of existing international codes. Indeed, the provision of improved international IIT codes is paramount to accurately and unambiguously assess the final quality, integrity, and performance of the large spectrum of manufactured products and engineering materials in an non-destructive manner. As the number of industrial activities and sectors involved in such an assessment process has been forecast to be quite large, the relative impact on the improved materials, products, processes, and systems and, in turn on our lives, safety, economy, environment, science, and technological progress is expected to be enormous and of great importance.
